# Supplementary material for: A tridimensional framework for governance in the wildland-urban interface using Pyro-Socio-Ecological Zones
Source: Nat Commun. 2025 Dec 18;16:11232. doi: 10.1038/s41467-025-66452-x (PMC12714711; doi:10.1038/s41467-025-66452-x)
Supplement: Supplementary file 1 — Supplementary Information [file 41467_2025_66452_MOESM1_ESM.pdf]

1. Supplementary Information

Supplementary Information Table 1. Dataset description for the Pyro-Socio-Ecological Zone typologies in Italy and California. (WUI: Wildland-Urban Interface; CAL FIRE FRAP: California Fire Alliance, Fire and Resource Assessment Program; ISPRA: Higher Institute for Environmental Protection and Research; NLCD: National Land Cover Database). Data descriptions were written and summarized by the authors based on the official documentation and metadata from each source.

**Italy**		**California**	
**Dataset name**	**Description**	**Dataset name**	**Description**
WUI dataset, from D'Este et al. (2021)[1]	The WUI consists of 9 classes and are the combination of the three categories of building types (isolated, scattered, and clustered) and three categories of tree canopy cover (low, medium and high).	WUI dataset from CAL FIRE FRAP[2,3]	The WUI comprises two distinct classifications, delineated based on building density across different territorial zones. The Wildland Urban *Interface* (WUI) denotes densely populated areas adjacent to vegetation susceptible to wildfires. Conversely, the Wildland Urban *Intermix* refers to residential developments situated within regions predominantly covered by wildland vegetation.

National Land Cover and Use Map - ISPRA[4]	Raster format with a spatial resolution of 10 meters, data employs a land use and cover classification system in accordance with the European guidance provided by the EAGLE Group and the MAES classification system. The latest land use map available dates back to 2022.	National Land Cover Data (NLCD)[5]	Nationwide data on land cover and its changes are available at a 30-meter resolution through the Landsat Thematic Mapper (TM). The most recent edition of this dataset is the NLCD 2021 from the U.S. Geological Survey (USGS) in collaboration with the Multi-Resolution Land Characteristics Consortium (MRLC).

Supplementary Information Table 2a. Defined housing density classes for the California 'Wildland Urban Interface 2019' dataset.

**Housing Density Class**	**Description**
1	Less than one house per 80937.1 m^2
2	One house per 80937.1 m^2 to one house per 20234.3 m^2
3	More than one house per 20234.3 m^2 to 1 house per acre
4	More than 1 house per 4046.86 m^2

Supplementary Information Table 2b. Wildland-Urban Interface (WUI) Assessment Table,' categorizing data into two sections: 'Interface WUI' and 'Intermix WUI.' It includes information on fire hazard severity zones, vegetation cover and spatial dimension.

Category	Housing Density Class	Fire Hazard Severity Zone	Vegetation	Spatial Dimension
Wildland Urban Interface	2, 3, 4	Moderate, High, Very High	Not dominated by wildland vegetation (not herbaceous, hardwood, conifer, or shrub)	Spatially contiguous groups of 30 m cells that are 40468.6 m^2 and larger
Wildland Urban Intermix	2	Moderate, High, Very High	Dominated by wildland vegetation; Improved parcels only	Spatially contiguous groups of 30 m cells 121406 m^2 and larger

Supplementary Information Table 3. Wildland-Urban Interface (WUI) Classification Table for Italy, categorizing nine WUI classes based on building count and Tree Canopy Cover. Each WUI class is assigned a unique code for identification.

Code	Class	Number of Buildings	Tree Canopy Cover
IB-LTC	Isolated buildings and low tree cover	<= 3	0–64%
IB-MTC	Isolated buildings and medium tree cover	<= 3	65–89%
IB-HTC	Isolated buildings and high tree cover	<= 3	90–100%
SB-LTC	Scattered buildings and low tree cover	4-49	0–64%
SB-MTC	Scattered buildings and medium tree cover	4-49	65–89%
SB-HTC	Scattered buildings and high tree cover	4-49	90–100%

CB-LTC	Clustered buildings and low tree cover	>= 50	0–64%
CB-MTC	Clustered buildings and medium tree cover	>= 50	65–89%
CB-HTC	Clustered buildings and high tree cover	>= 50	90–100%

Supplementary Information Table 4. Scale of Human Development Index (S) Estimation for Italy and California.

**Italy**		**California**	
Municipal (km^2)		US Census Tract (km^2)	
Minimum	Maximum	Minimum	Maximum
0.07	590.99	0.06	3024.62

Supplementary Information Table 5. Scale of Governance Indicators (G) Estimation for Italy and California.

**Italy**		**California**	
Municipal (km^2)		Municipal (km^2)	
Minimum	Maximum	Minimum	Maximum
0.07	590.99	0.08	1302.06

Supplementary Information Table 6. Datasets summary of Governance Quality Indicators. (ISTAT: Italian National Institute of Statistics; INPS: National Institute of Social Security; UCR: Uniform Crime Reporting; CDSS: California Department of Social Services)

**Indicator**	**Italy datasets**	**California datasets**
Transparency	Average number of corruption cases (Source:	Average number of corruption cases (Source: CalMatters[7], Voice of OC[8],

	Transparency International Italia[6])	The Desert Sun[9], PBS News[10], FOX40 News[11], KQED[12], ACLU of Southern California[13])
Participation	Rate of participation in elections (Source: ISTAT[14])	Rate of participation in elections (Source: Secretary of State – California[15])
Equity	Euro per capita for home-based, residential and semi-residential social assistance, transportation of the disabled and elderly and Euro per capita of municipalities' social spending on children, the disabled and the elderly (Source: ISTAT[14], INPS[16], Chamber of Commerce Study Center[17])	Net expenditures for homeless assistance, the annual report on CalWORKs beneficiaries, Foster Care, social services, Non-Assistance CalFresh, Welfare to Work, Refugee Cash Assistance, and the Cash Assistance Program for Immigrants (Source: CDSS[18])
Legality	Index of Permeability to Organized Crime (Source: Eurispes[19])	Total number of reports of gang-related activities (Source: CalGang[20])
Safety	Number of reports of crime occurrence per 100 thousand inhabitants (Source: ISTAT[14], Police Forces[21])	Number of reports of crime occurrence per 100 thousand inhabitants (Source: UCR[22])
Environmental Regulatory Quality	Presence/absence of environmental laws (Source: Regional Forestry Law)	Presence/absence of environmental laws (Source: Sites of counties, departments and cities)

Supplementary Information Table 7. Summary of land cover categories in Italy and California.

Italy	Land Cover Categories	California
Forests	Forest	Deciduous Forest
Wetlands		Evergreen Forest
		Mixed Forest
		Woody Wetlands
Urban and assimilated areas	Developed	Developed: Open Space
		Developed: Low Intensity
		Developed: Medium Intensity
		Developed: High Intensity
Permanent crops	Agriculture Areas	Cultivated Crops
Seed crops		Pasture/Hay
Forage crops		
Agroforestry areas		
Other agricultural areas		
Other non-economic uses	Other Natural Areas	Shrub/Scrub
		Grassland/Herbaceous
		Emergent Herbaceous Wetlands
Water uses	Other	Open Water
Quarries and mines		Perennial Ice and Snow
NA		Barren Land
		NA

Supplementary Information Table 8. Land Cover Types and Weighting Criteria for the Ecological Management dimension.

Land Cover types	Weight	References

Forest	1	[23–25]
Other Natural Areas	0.75	[26–29]
Agriculture Areas	0.50	[30–32]
Other	0.25	[33]
Developed	0	[34–36]

Supplementary Information Table 9. Dataset summary for determining the quantity and type of biomass within new type Wildland-Urban Interface (WUI) areas in Italy and California. Note, GEDI: Global Ecosystem Dynamics Investigation; INFC: National Inventory of Forests and Forest Carbon Sinks; CMS: Carbon Monitoring System; FIA: Forest Inventory and Analysis.

**Italy**		**California**	
**Dataset name**	**Description**	**Dataset name**	**Description**
GEDI[37]	Generates detailed three-dimensional maps of forest canopies using advanced laser-ranging technology aboard the International Space Station (ISS). This dataset was updated in August 2021.	GEDI[37]	Generates detailed three-dimensional maps of forest canopies using advanced laser-ranging technology aboard the International Space Station (ISS). This dataset was updated in August 2021.
INFC[38]	Quantitative data on the stand (number of trees, basal area, volume, increase, etc.); values per	CMS from FIA[39]	Estimates include the mean (and standard error of the mean) biomass of live trees, calculated using three

	inventory test area, per hectare.		sets of allometric equations. The estimated forest area is on a hexagonal plot of 650 km^2.

Supplementary Information Table 10. Color-coded Matrix of Pyro-Socio-Ecological Zones (PSEZ) with color and Hex Code (a six-digit hexadecimal code precisely defines colors in digital formats).

**Ecological (E)**	**Socioeconomic (S)**	**Governance (G)**	**Color**	**Hex Code**
Low	Low	Low	Black	#000000
Low	Low	Medium	Blue	#0000AA
Low	Low	High	Deep Blue	#0020FF
Low	Medium	Low	Green	#00AA00
Low	Medium	Medium	Aqua	#00AAAA
Low	Medium	High	Sky Blue	#00AAFE
Low	High	Low	Bright Green	#00FF00
Low	High	Medium	Mint Green	#00FFAA
Low	High	High	Light Cyan	#00EFFF
Medium	Low	Low	Dark Red	#AA0000
Medium	Low	Medium	Purple	#AA00AA
Medium	Low	High	Lavender	#AA50FF
Medium	Medium	Low	Yellow	#AAAA00
Medium	Medium	Medium	Gray	#AAAAAA
Medium	Medium	High	Light Green	#AAFFAA
Medium	High	Low	Forest Green	#55AA55
Medium	High	Medium	Light Blue	#55AAFF
Medium	High	High	Pale Cyan	#99FFEE
High	Low	Low	Bright Red	#FF0000

High	Low	Medium	Magenta	#FF00AA
High	Low	High	Pink	#FF75EE
High	Medium	Low	Orange	#FFAA00
High	Medium	Medium	Light Pink	#FFAAAA
High	Medium	High	Pale Pink	#FFCCFF
High	High	Low	Yellow	#FEEE00
High	High	Medium	Pale Yellow	#FFFF99
High	High	High	Off White	#FFFFEE

1.1. Statistical and spatial analysis

We developed the PSEZ by using ArcGIS Pro for all spatial analyses, which involved multiple steps. First, the Extract by Mask function was applied to filter the NLCD and GEDI biomass datasets using WUI masks, isolating relevant spatial features. Second, raster data, including land cover and biomass, were converted into polygon features to facilitate spatial analysis and intersection with other datasets. Then, spatial intersections were performed between WUI areas and county and region boundaries, integrating demographic, economic and administrative information. Third, attribute joins were used to merge WUI data with external datasets, enhancing the attribute table with additional information. Intersections between WUI categories and NLCD/CLC classes were tabulated to create a comprehensive dataset detailing land cover types within the WUI. Fourth, multiple datasets were merged to create integrated spatial layers, encompassing various aspects of land use and biomass distribution. Finally, the GEDI biomass data was resampled to ensure consistency with other spatial datasets (National Forest Inventories) in terms of resolution and alignment. These geoprocessing steps enabled a detailed analysis of WUI areas, providing insights into land cover, vegetation biomass distribution, and the spatial distribution of socioeconomic and governance.

All statistical analyses were performed in R version 4.1.2 software [40]. Package 'dplyr' was employed for data manipulation (e.g., filtering rows, selecting columns, creating new variables, grouping data by specific attributes, and summarizing with mean values), including sum and percentage calculations for each PSEZ class over different geographic units (ecoregion, county, region) and WUI type. The results were visualized through stacked bar graphs, created with 'ggplot2', to represent the distribution of PSEZ classes by county, ecoregion, WUI type, and region, with color specification for each land class to ensure uniformity across graphs.

1.2. Justification of governance index methods

Our study areas in southern Italy and California face unique challenges due to their geographical and socioeconomic conditions and as a result, require context-specific governance strategies[41,42]. Most importantly, given their Mediterranean climate, these landscapes are especially susceptible to wildfire risk because of arid, hot summers, their biodiversity, and many of the same anthropogenic pressures and extreme climate event-related challenges[43]. However, applying the same and existing WUI typologies to southern Italy and California[44] fails to capture their unique socio-political and cultural diversity and management challenges related to fire risk[45]. More specifically, both regions experience similar urban development and environmental preservation issues, but they differ in governance frameworks and socio-ecological contexts[46–48]. For example, California relies on federal guidelines and localized assessments to guide fire risk mapping strategies, whereas Italy's decentralized governance results in a range of regional definitions and regulations[49]. Billings et al. (2021)[50] also found that trust in government influenced resident support for incentives and aid in implementing fire risk mitigation measures in the WUI.

Therefore, spatially explicit indices and mapping approaches could assist in designing more effective conservation, strong governance, and sustainable stewardship practices[51,52]. But socioeconomic and governance indicators need to be adapted to reflect localized contexts and conditions particularly at the scale of human settlements such as communities and municipalities[53,54] Accordingly, we adapted the Human Development Index[55] and the Worldwide Governance Indicators[56] to our framework. Both indices have been used internationally across different socio-political scales by organizations such as The United Nations and The World Bank to account for not only human development and wellbeing but also experts and citizen perception regarding the quality of governance. Section 2.3 in the main text details how they were both adapted into our two study areas and PSEZ framework.

2. References

1. D’Este, M., Giannico, V., Lafortezza, R., Sanesi, G. & Elia, M. The wildland-urban interface map of Italy: A nationwide dataset for wildfire risk management. *Data in Brief* **38**, 107427 (2021).
2. CAL FIRE FRAP, Fire and Resource Assessment Program - California Department of Forestry and Fire Protection & State of California. Dataset: Wildland Urban Interface 2019. (2019).

3. California Fire Alliance, Sacramento, California, USA. California Fire Alliance. 2001. Characterizing the fire threat to wildland-urban interface. (2001).
4. ISPRA. National Land Cover and Use Map. (2022).
5. U.S. Geological Survey, (https://www.usgs.gov/centers/eros/science/national-land-cover-database). National Land Cover Database.
6. Transparency International Italia, (https://transparency.it/). Average number of corruption cases.
7. CalMatters, (https://calmatters.org/). Average number of corruption cases.
8. Voice of OC, (https://voiceofoc.org/). Average number of corruption cases.
9. Desert Sun, (https://eu.desertsun.com/). Average number of corruption cases.
10. PSB, (https://www.pbs.org/). Average number of corruption cases.
11. FOX40, (https://fox40.com/). Average number of corruption cases.
12. KQED, (https://www.kqed.org/). Average number of corruption cases.
13. ACLU of Southern California, (https://www.aclusocal.org/). Average number of corruption cases.
14. Italian National Institute of Statistics (ISTAT), (https://www.istat.it/). General censuses of population, services and industry, agriculture, household sample surveys and general economic surveys at the national level.
15. California Secretary of State, (https://www.sos.ca.gov/). Rate of participation in elections.
16. National Institute of Social Security (INPS), (https://www.inps.it/it/it/dati-e-bilanci/open-data.html). Pension institution data of the Italian public pension system.
17. Chamber of Commerce Study Center, (https://www.unioncamere.gov.it/osservatori-economici/centro-studi). Analysis and monitoring of socio-economic phenomena in Italy.
18. California Department of Social Services (CDSS), (https://www.cdss.ca.gov/inforesources/data-portal). Data designed to inform the public about the social services programs and policies of California residents each year.
19. Eurispes - The Research Institute of Italians, (https://eurispes.eu/). Italian private entity concerned with political, economic and social studies, and working in the field of political, economic, social and educational research.

20. CalGang® | State of California - Department of Justice, (https://oag.ca.gov/calgang). Total number of reports of gang-related activities.
21. Polizia di Stato Italia, (https://www.poliziadistato.it/). Number of reports of crime occurrence per 100 thousand inhabitants.
22. Uniform Crime Reports (UCR), (https://www.fbi.gov/how-we-can-help-you/more-fbi-services-and-information/ucr). Crime/Law Enforcement Stats.
23. Msofe, N. K., Sheng, L., Li, Z. & Lyimo, J. Impact of Land Use/Cover Change on Ecosystem Service Values in the Kilombero Valley Floodplain, Southeastern Tanzania. *Forests* **11**, 109 (2020).
24. Solomon, N., Segnon, A. C. & Birhane, E. Ecosystem Service Values Changes in Response to Land-Use/Land-Cover Dynamics in Dry Afromontane Forest in Northern Ethiopia. *IJERPH* **16**, 4653 (2019).
25. Spanò, M., Leronni, V., Lafortezza, R. & Gentile, F. Are ecosystem service hotspots located in protected areas? Results from a study in Southern Italy. *Environmental Science & Policy* **73**, 52–60 (2017).
26. Bisui, S., Roy, S., Sengupta, D., Bhunia, G. S. & Kumar Shit, P. Assessment of ecosystem services values in response to land use/land cover change in tropical forest. in *Forest Resources Resilience and Conflicts* 435–447 (Elsevier, 2021). doi:10.1016/B978-0-12-822931-6.00031-9.
27. Sala, O. E. & Maestre, F. T. Grass–woodland transitions: determinants and consequences for ecosystem functioning and provisioning of services. *Journal of Ecology* **102**, 1357–1362 (2014).
28. Yoshida, A., Chanhda, H., Ye, Y.-M. & Liang, Y.-R. Ecosystem service values and land use change in the opium poppy cultivation region in Northern Part of Lao PDR. *Acta Ecologica Sinica* **30**, 56–61 (2010).
29. Zhao, Y., Liu, Z. & Wu, J. Grassland ecosystem services: a systematic review of research advances and future directions. *Landscape Ecol* **35**, 793–814 (2020).
30. Byrd, K. B. *et al.* Integrated climate and land use change scenarios for California rangeland ecosystem services: wildlife habitat, soil carbon, and water supply. *Landscape Ecol* **30**, 729–750 (2015).
31. Geneletti, D. An approach based on spatial multicriteria analysis to map the nature conservation value of agricultural land. *Journal of Environmental Management* **83**, 228–235 (2007).

32. Jiang, L., Wang, Z., Zuo, Q. & Du, H. Simulating the impact of land use change on ecosystem services in agricultural production areas with multiple scenarios considering ecosystem service richness. *Journal of Cleaner Production* **397**, 136485 (2023).
33. Courault, R. & Cohen, M. Evolution of Land Cover and Ecosystem Services in the Frame of Pastoral Functional Categories: A Case Study in Swedish Lapland. *Sustainability* **12**, 390 (2020).
34. Liu, W. *et al.* Impacts of urbanization-induced land-use changes on ecosystem services: A case study of the Pearl River Delta Metropolitan Region, China. *Ecological Indicators* **98**, 228–238 (2019).
35. McDonald, R. I., Marcotullio, P. J. & Güneralp, B. Urbanization and global trends in biodiversity and ecosystem services. *Urbanization, biodiversity and ecosystem services: challenges and opportunities: a global assessment* 31–52 (2013).
36. Peng, J. *et al.* Ecosystem services response to urbanization in metropolitan areas: Thresholds identification. *Science of The Total Environment* **607–608**, 706–714 (2017).
37. Dubayah, R. O. *et al.* Global Ecosystem Dynamics Investigation (GEDI)GEDI L4B Gridded Aboveground Biomass Density, Version 2. 0 MB (2022) doi:10.3334/ORNLDAAC/2017.
38. Arma dei Carabinieri, Consiglio per la ricerca in agricoltura e l'analisi dell'economia agraria. Inventario Nazionale delle Foreste e dei serbatoi forestali di Carbonio – INFC. (2022).
39. Menlove, J. & Healey, S. P. Carbon Monitoring System (CMS)CMS: Forest Aboveground Biomass from FIA Plots across the Conterminous USA, 2009-2019. 0 MB (2021) doi:10.3334/ORNLDAAC/1873.
40. R Core Team. *R: A Language and Environment for Statistical Computing*. (R Foundation for Statistical Computing, Vienna, Austria, 2021).
41. Bar-Massada, A., Alcasena, F., Schug, F. & Radeloff, V. C. The wildland – urban interface in Europe: Spatial patterns and associations with socioeconomic and demographic variables. *Landscape and Urban Planning* **235**, 104759 (2023).
42. Cheng, H., Escobedo, F. J., Thomas, A. S., De Los Reyes, J. F. & Soto, J. R. Comparing individual and collective valuation of ecosystem service tradeoffs: A case study from montane forests in southern California, USA. *Ecosystem Services* **69**, 101648 (2024).

43. Hamilton, M., Fischer, A. P. & Ager, A. A social-ecological network approach for understanding wildfire risk governance. *Global Environmental Change* **54**, 113–123 (2019).

44. Radeloff, V. C. *et al.* THE WILDLAND–URBAN INTERFACE IN THE UNITED STATES. *Ecological Applications* **15**, 799–805 (2005).

45. Schug, F. *et al.* The global wildland–urban interface. *Nature* **621**, 94–99 (2023).

46. Coluzzi, R. *et al.* Density matters? Settlement expansion and land degradation in Peri-urban and rural districts of Italy. *Environmental Impact Assessment Review* **92**, 106703 (2022).

47. Wei, Y. D. & Ewing, R. Urban expansion, sprawl and inequality. *Landscape and urban planning* **177**, 259–265 (2018).

48. Zambon, I., Benedetti, A., Ferrara, C. & Salvati, L. Soil Matters? A Multivariate Analysis of Socioeconomic Constraints to Urban Expansion in Mediterranean Europe. *Ecological Economics* **146**, 173–183 (2018).

49. Corona, P. *et al.* Elementi di orientamento per la pianificazione forestale alla luce del Testo Unico in materia di foreste e filiere forestali. *Rete Rurale Nazionale* **2020**, (2014).

50. Billings, M., Carroll, M., Paveglio, T. & Whitman, K. “Us versus Them;” Local Social Fragmentation and Its Potential Effects on Building Pathways to Adapting to Wildfire. *Fire* **4**, 96 (2021).

51. Loveland, T. R. & Merchant, J. M. Ecoregions and Ecoregionalization: Geographical and Ecological Perspectives. *Environmental Management* **34**, S1–S13 (2004).

52. Wellman, J. D. & Tipple, T. J. Governance in the wildland-urban interface: a normative guide for natural resource managers. in *Culture, Conflict, And Communication In The Wildland-urban Interface* 337–347 (Routledge, 2019).

53. Thomas, M. A. What Do the Worldwide Governance Indicators Measure? *Eur J Dev Res* **22**, 31–54 (2010).

54. Hayes, T. & Murtinho, F. Communal governance, equity and payment for ecosystem services. *Land Use Policy* **79**, 123–136 (2018).

55. Sheth, S. & Bettencourt, L. M. A. The Community Human Development Index (CHDI): Localizing Sustainable Development Goals Across Scales. in *2020 IEEE Conference on Technologies for*

Sustainability (SusTech) 1–7 (IEEE, Santa Ana, CA, USA, 2020). doi:10.1109/SusTech47890.2020.9150510.

56. Keser, A. & Gökmen, Y. Governance and Human Development: The Impacts of Governance Indicators on Human Development. *JPAG* **8**, 26 (2018).
